# Suppression of NANOG Expression Reduces Drug Resistance of Cancer Stem Cells in Glioblastoma

**DOI:** 10.3390/genes14061276

**Published:** 2023-06-16

**Authors:** Jonhoi Smith, Melvin Field, Kiminobu Sugaya

**Affiliations:** 1 Burnett School of Biomedical Sciences, College of Medicine, University of Central Florida, Orlando, FL 32816, USA; jsmith100@knights.ucf.edu (J.S.); dr.field@orlandoneurosurgery.com (M.F.); 2 Orlando Neurosurgery, AdventHealth Neuroscience Institute, Orlando, FL 32803, USA

**Keywords:** cancer stem cells, glioblastoma, stemness genes, NANOG, OCT4, RNAi, TMZ, PI3K/AKT

## Abstract

Glioblastoma (GBM) is an aggressive and incurable primary brain tumor that harbors therapy-resistant cancer stem cells (CSCs). Due to the limited effectiveness of conventional chemotherapies and radiation treatments against CSCs, there is a critical need for the development of innovative therapeutic approaches. Our previous research revealed the significant expression of embryonic stemness genes, NANOG and OCT4, in CSCs, suggesting their role in enhancing cancer-specific stemness and drug resistance. In our current study, we employed RNA interference (RNAi) to suppress the expression of these genes and observed an increased susceptibility of CSCs to the anticancer drug, temozolomide (TMZ). Suppression of NANOG expression induced cell cycle arrest in CSCs, specifically in the G0 phase, and it concomitantly decreased the expression of PDK1. Since PDK1 activates the PI3K/AKT pathway to promote cell proliferation and survival, our findings suggest that NANOG contributes to chemotherapy resistance in CSCs through PI3K/AKT pathway activation. Therefore, the combination of TMZ treatment with RNAi targeting NANOG holds promise as a therapeutic strategy for GBM.

## 1. Introduction

Glioblastoma (GBM), a grade IV astrocytoma, is the most common and highly malignant central nervous system (CNS) tumor that currently lacks adequate treatment [1]. The cerebral hemispheres, specifically the supratentorial region, are the prevalent location for GBM tumors [2]. For newly diagnosed patients, the standard treatment regimen starts with a maximal surgical resection of the tumor tissue followed by radiation and chemotherapy [3,4]. However, the median life expectancy of GBM is only 12–16 months with the currently available therapies [5]. The poor prognosis of GBM can be attributed to the heterogenetic nature of the disease, which means that it exhibits a wide range of genetic and molecular characteristics. A major factor contributing to the treatment resistance of GBM is the presence of cancer stem cells (CSCs).

CSCs were first discovered in acute myeloid leukemia [6], and they were later identified in cancer within the brain, heart, breast, colon, and lung [7,8,9,10]. They possess self-renewal and high-differentiation capabilities, which are characteristics of stem cells [11,12], and they play a pivotal role in cancer progression, facilitating uncontrolled cell proliferation, metastasis, and the initiation of new tumor formation [13,14]. CSCs can be distinguished from other cells by utilizing cell surface markers commonly found on stem cells, such as CD24, CD29, CD44, and CD133 [15,16]. The drug resistance of CD133-positive cells has been confirmed in various types of cancers, including GBM [17,18,19,20].

The resistance of CSCs against temozolomide (TMZ), the current standard therapy for GBM, has been reported [21,22]. Temozolomide is a small lipophilic molecule that can effectively cross the blood–brain barrier (BBB) [23]. It functions as a DNA-alkylating agent, adding a methyl group to the purine bases of DNA. This methylation process leads to the formation of cytotoxic mispairings with pyrimidine bases [24]. By inducing these abnormal DNA structures, TMZ disrupts DNA replication and ultimately triggers cell death [25]. While the exact mechanism is not fully understood, the resistance of CSCs to TMZ is attributed to their stemness properties [21,22].

Previous studies have indicated that stemness genes contribute to an increase in the expression of O^6^-methylguanine-DNA methyltransferase (MGMT), an enzyme involved in DNA repair that can counteract the effects of TMZ [26,27,28]. Elevated levels of MGMT are frequently observed in GBM patients, and epigenetic modifications of the MGMT promoter are commonly employed to predict the responsiveness of GBM patients to TMZ treatment [29,30]. MGMT levels are higher in CSCs [18], and there have been reports of a correlation between the presence of CSCs in tumor tissue and the expression of MGMT [31].

We have previously reported that embryonic stemness genes, specifically NANOG and OCT4, which are members of the DNA-binding homeobox transcription factor family, exhibit a higher expression in CSCs compared to normal neural stem cells and other cancer cells in GBM [32]. This finding has been supported by independent researchers [33]. Recently, we found that CSC-secreted exosomes contain the NANOG family retro-oncogene NANOGP8 with a distinct promoter [34]. These exosomes, enriched with stemness genes, possessed the ability to induce a transformation of GBM cancer cells into a cancer-specific stemness phenotype [26]. Considering these discoveries and the well-established association between the expression of stemness genes and drug resistance in various cancers [22,35,36,37,38], it is plausible that embryonic stemness genes play a critical role in sustaining the cancer-specific stemness of GBM and contribute to the observed drug resistance in CSCs [39,40,41]. Therefore, in our current research, we employed RNA interference (RNAi) to suppress the expression of stemness genes in CSCs, aiming to investigate whether the downregulation of stemness genes increases the vulnerability to TMZ.

Furthermore, in addition to the developmental pathways such as JAK/STAT, Hedgehog, Wnt, and NOTCH, which have been reported to be crucial for maintaining the stemness of CSCs [42,43], the PI3K/AKT pathway plays a significant role in regulating proliferation, metabolism, and cell survival following the activation of the receptor tyrosine kinase, phosphoinositide-3-kinase (PI3K). The dysregulation of this pathway is frequently observed in cancer and may potentially impact the response to therapy [44]. Studies have indicated that elevated PI3K/AKT signaling is associated with the expression of stemness genes in CSCs [45]. In light of these findings, we conducted additional investigations to explore the involvement of the PI3K/AKT signaling pathway in the development of drug resistance in CSCs.

## 2. Materials and Methods

### 2.1. Cell Culture

Primary glioblastoma cells were obtained from tumor tissue excised during surgery from a patient with GBM, following approved protocols from Advent Health Hospital and the University of Central Florida Institutional Review Boards. Informed consent was obtained from the subjects, and compliance with Health Insurance Portability and Accountability Act (HIPAA) regulations was strictly adhered to. The cells were cultured as spheroids in human neural stem cell (HNSC) media, which comprised Dulbecco’s modified eagle medium/nutrient mixture F-12 (DMEM/F12) supplemented with heparin (0.5 U/mL), epidermal growth factor (EGF) (20 ng/mL), basic fibroblast growth factor (bFGF) (20 ng/mL), and 2% B27. The cultures were maintained at 37 °C with 5% CO_2_. Once the spheroids reached a diameter of approximately 1 mm, they were dissociated using Accutase (Gibco™ StemPro™ Accutase™ Cell Dissociation Reagent, catalog #A1110501), washed with phosphate-buffered saline (PBS), and suspended in 300 µL of PBS. Subsequently, CD133^+^ and CD133^−^ cells were separated from the cell suspension using CD133-antibody-conjugated magnetic microbeads (Miltenyi Biotec, CD133 microbeads, human) following the manufacturer’s protocol. The CD133^+^ cells were cultured in the aforementioned HNSC media for further proliferation, while the CD133^−^ cells were cultured in DMEM/F12 media supplemented with 10% fetal bovine serum (FBS). HEK 293TN cells (SBI catalog number: LV900A-1) were cultured as a monolayer in DMEM media supplemented with 10% FBS.

### 2.2. Production of Lentivirus Containing shRNA

Lentiviral vectors were employed to deliver short hairpin RNA (shRNA) targeting NANOG (shRNA NANOG: target sequence AGATGAGTGAAACTGATATTA) and OCT4 (shRNA OCT4: target sequence AGAAGTCCCAGGACATCAAAG). These lentiviral vectors, with H1-promoter-driven shRNA gene cassettes, were custom-made by VectorBuilder (Chicago, IL, USA) with the following vector IDs: VB160620-1054sjk (NANOG) and VB160620-1053cvf (OCT4). HEK 293TN cells were seeded at 75–90% confluency for lentiviral particle production. The packaging, envelope, and transfer plasmids carrying the shRNA targeting NANOG or OCT4 were co-transfected into HEK293TN cells using Lipofectamine™ 2000 (ThermoFisher, Waltham, MA, USA, Catalog number: 11668019). Transfection efficacy was determined by the number of fluorescent-positive colonies observed the day following the transfection. Spent media containing viral particles were collected over the next three days. The viral-containing media were stored at 4 °C until viral isolation. The viral particles were precipitated from the spent cell culture media using PEG-it 5× virus precipitation solution (SBI) following the manufacturer’s protocol. Subsequently, the viral particles were resuspended in PBS, aliquoted, and stored at −80 °C until further use.

### 2.3. Lentiviral Transduction

Prior to viral transduction, CD133^+^ GBM spheroids were carefully dissociated using Accutase to obtain single-cell suspensions. The cells were then allowed to recover for several hours before being transferred to a 6-well plate containing HNSC media supplemented with polybrene (4 μg/mL). Lentivirus was added to the cell cultures, and they were incubated overnight. Successful transduction was confirmed by the expression of mCherry, which was observed under a fluorescent microscope. To select for shRNA-positive cells, the cells were cultured in HNSC media supplemented with 400 µg/mL of geneticin. For long-term maintenance of the culture, the concentration of geneticin was subsequently lowered to 200 µg/mL.

### 2.4. Two-Step Quanitative Reverse Transcription-PCR

The cells were lysed using TRIzol reagent (Invitrogen, Waltham, MA, USA, Cat: 15596026), and RNA was extracted following the manufacturer’s protocol using a Direct-zol RNA kit (Zymo Research, Los Angeles, MA, USA, Cat: R2061). Subsequently, 0.5 µg of RNA was utilized to synthesize cDNA with an AzuraQuantTM cDNA Synthesis Kit (Azura genomics, Raynham, MA, USA, Cat: AZ-1995). For qPCR analysis, 20 µL reactions were set up using specific primer pairs (Table 1) and either AzuraQuant™ Green Fast qPCR Mix LoRox (Azura genomics, Raynham, MA, USA, Cat: AZ-2101) or Applied Biosystems™ SYBR™ Green PCR Master Mix (ThermoFisher, Waltham, MA, USA) (Cat: 4385612). The reactions were performed using the QuantStudio 7 Flex real-time PCR system (Applied Biosystems, Waltham, MA, USA) with the following cycling conditions: initial denaturation at 95 °C for 20 s, followed by 40 cycles of denaturation at 95 °C for 1 s, and annealing/extension at 60 °C for 20 s. The relative gene expressions were analyzed using the 2^−ΔΔCT^ method.

### 2.5. Cell Viability Assay

CD133^+^ cells were seeded at a density of 5000 cells per well in a 96-well plate. The cells were treated with TMZ (TCI chemicals, Portland, OR, USA, Cat: T2744) for 24 h at concentrations ranging from 10 µM to 1 mM. TMZ solutions were prepared by 1/10 serial dilutions of a 100 mM stock solution in 100% DMSO. The experiment was performed in triplicate. To evaluate cell viability, ethidium homodimer III (EthD-III) (Invitrogen, Waltham, MA, USA, Cat: L3224), a nucleic acid dye that specifically stains dead cells while live cells remain unstained, was used. The fluorescence signal from EthD-III was measured using an EnVision 2104 Multilabel Reader (PerkinElmer, Waltham, MA, USA). The combination effect of the TMZ and shRNA treatments was analyzed based on the principles described by Chou and Talalay [46]. The coefficient of drug interaction (CDI) was determined using the following formula: CDI = SAB/(SA × SB), where SAB represents the survival rate of the combination treatment of TMZ and shRNA relative to the control group, and SA and SB represent the survival rates of the TMZ and shRNA treatments, respectively, relative to the control group. CDI values less than 1, equal to 1, and greater than 1 indicated synergistic, additive, and antagonistic effects, respectively.

### 2.6. Cell Cycle Analysis

Cell cycle analysis was performed using flow cytometry [47,48]. CD133^+^ cells were seeded at a density of 500,000 cells per well in 12-well plates. Lentiviral stocks containing shRNA targeting NANOG were transduced into the cells as previously described, and the cells were allowed to rest for 2 days. Positive selection of shRNA targeting NANOG cells was conducted using HNSC media supplemented with geneticin (400 µg/mL) for 5–7 days. After selection, the cells were dissociated into a single-cell suspension using Accutase and washed twice with PBS. The cells were fixed by adding 500 µL of cold 70% ethanol dropwise to the cell suspension and incubated on ice for 1 h. Subsequently, the cells were stained with 300 µL of PI solution and incubated at 37 °C for 40 min. The stained cells were then analyzed for DNA content using CytoFlex (Beckman Coulter, Brea, CA, USA) for a duration of 30 min.

### 2.7. Statistical Analysis and Graphs

Statistical analysis and data visualization were conducted using GraphPad Prism 9 (GraphPad Software 9.3.1).

## 3. Results

### 3.1. Suppression of Stemness Genes NANOG and OCT4 Expressions by Short Hairpin RNA Interference (shRNA)

**Figure 1:**
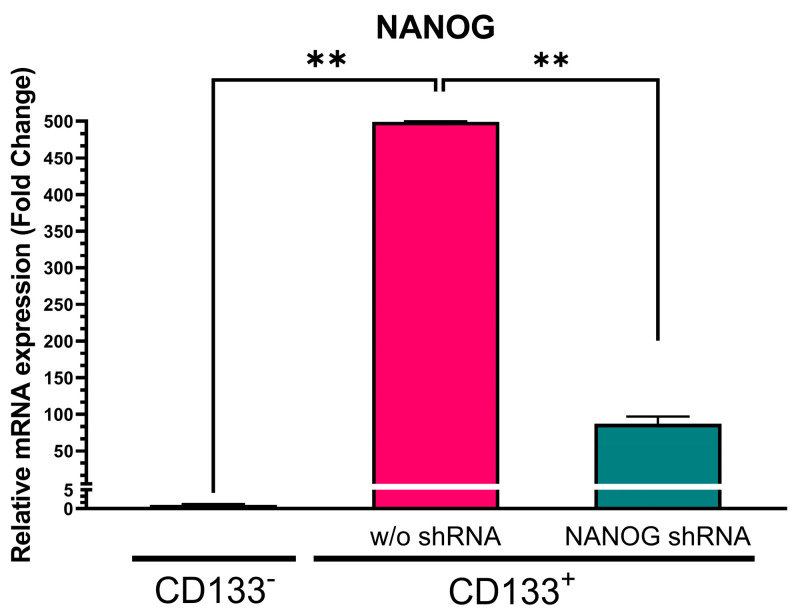
Relative NANOG mRNA expression in CD133^−^ GBM cells, CD133^+^ GBM cells, and CD133^+^ GBM cells treated with NANOG shRNA. Relative expression was normalized using a housekeeping gene, β-actin, as an internal standard. The relative gene expression level was expressed as fold changes compared to the gene expression level in CD133^−^ cells. The statistical analysis was performed with a one-way ANOVA followed by the Holm–Sidaks multiple comparison test. ** *p* < 0.01.

**Figure 2:**
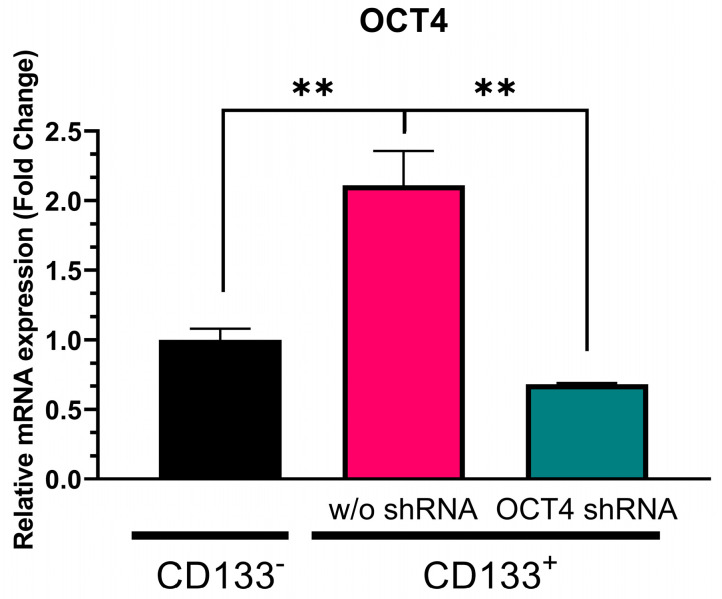
Relative OCT4 mRNA expression in CD133^−^ GBM cells, CD133^+^ GBM cells, and CD133^+^ cells treated with OCT4 shRNA. Relative expression was normalized using a housekeeping gene, β-actin, as an internal standard. The relative gene expression level was expressed as fold changes compared to the gene expression level in CD133^−^ cells. The statistical analysis was performed with a one-way ANOVA followed by the Holm–Sidaks multiple comparison test. ** *p* < 0.01.

We initially separated CSCs as floating spheroids from GBM-patient-derived tumor tissue using nonadherent cultures and purified them with CD133-antibody-conjugated magnetic beads. Therefore, the CD133^+^ cells were CSCs, and the CD133^−^ cells were non-CSCs. Figure 1 illustrates that NANOG expression in the CD133^+^ GBM cells was approximately 500-fold higher (*p* = 0.0005) than in the CD133^−^ GBM cells. To suppress NANOG mRNA expression, we designed a gene cassette driven by an H1 promoter that produced short hairpin RNA (shRNA) targeting the NANOG transcript. This shRNA construct was delivered to the cells using lentivirus. The introduction of NANOG shRNA resulted in a reduction of over 80% in NANOG expression (*p* = 0.0078) (Figure 1). Additionally, we investigated the suppression of OCT4, a transcriptional regulator that interacts with NANOG (52). We observed a significantly higher expression of OCT4 in the CD133^+^ GBM cells than in the CD133^−^ GBM cells (Figure 2). Upon delivery of the shRNA targeting OCT4 transcripts, OCT4 expression in the CD133^+^ GBM cells was reduced by more than 65% (*p* = 0.000023).

### 3.2. Reduction in Stemness Gene Expression Increased CSC Vulnerability against TMZ

**Figure 3:**
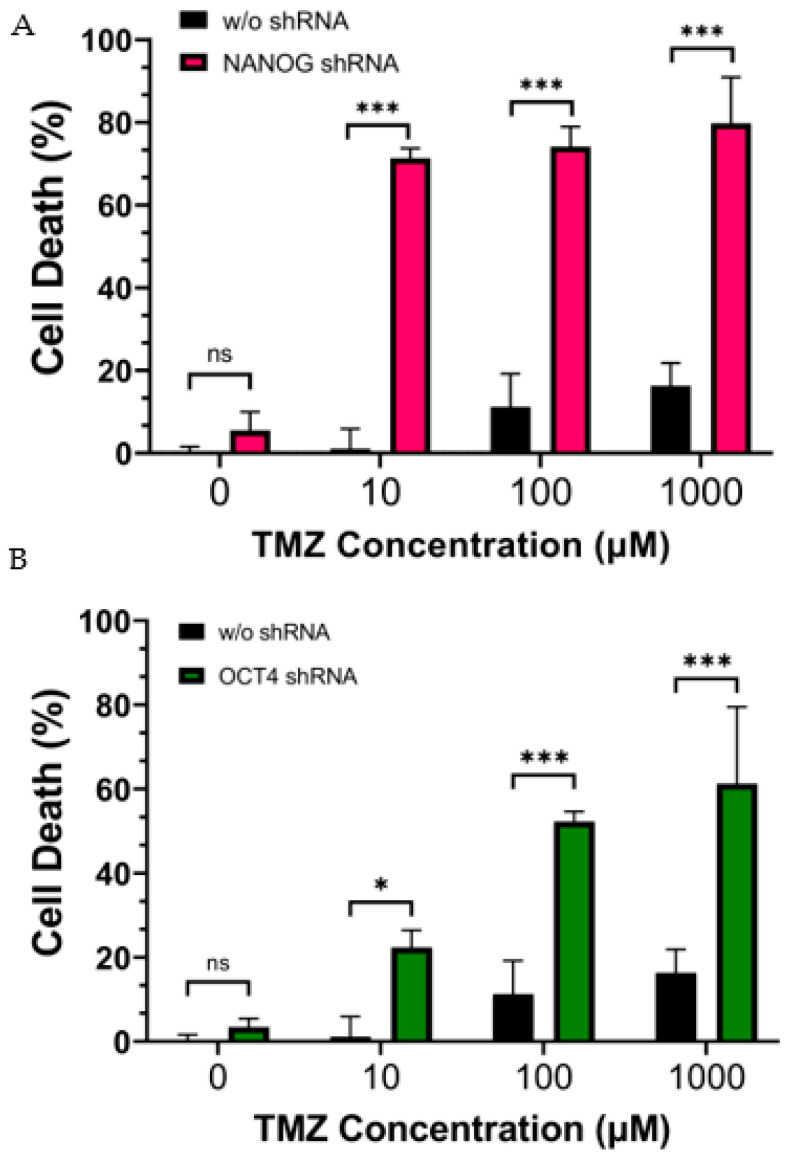
Cell death of CD133^+^ GBM cells after 24 h of treatment with TMZ (10 µM ∓ 1 mM). (**A**) shows the effect of the suppression of NANOG by shRNA. (**B**) shows the effect of the suppression of OCT4 by shRNA. Statistical analysis was performed using a two-way ANOVA followed by Fisher’s LSD test. ^ns^ Not significant, * *p* < 0.05, *** *p* < 0.001.

In order to investigate whether the reduction in stemness genes increases the sensitivity of CD133^+^ GBM to chemotherapy, we treated CD133^+^ GBM cells with varying concentrations of TMZ along with shRNA gene cassettes targeting NANOG or OCT4. Cell viability was assessed using red fluorescent nucleic-acid-binding ethidium homodimer III (EthD-III) dye, which penetrates cells with compromised plasma membranes, thus indicating cell death. When the CD133^+^ GBM cells were treated with TMZ alone, the intensity of cell death increased in a dose-dependent manner (Figure 3A,B). However, even at the highest concentration of TMZ (1 mM), less than 20% of the GBM cells were killed. When the CD133^+^ GBM cells were treated with shRNA targeting NANOG or OCT4 in combination with TMZ, the level of cell death was significantly higher compared to with the TMZ treatment alone, suggesting that the inhibition of NANOG or OCT4 increased the efficacy of TMZ. Furthermore, a dose-dependent response was observed when the TMZ treatment was combined with the shRNA treatment. We calculated the coefficient of drug interaction (CDI) to determine whether the combination treatment had an additive or synergistic effect on the CD133^+^ GBM cells. The CDI values were less than 1 for all the concentrations tested, as shown in Table 2. Therefore, the combination treatment with TMZ and the suppression of NANOG or OCT4 expression exhibited a synergistic effect, indicating different mechanisms of action between the two treatments.

### 3.3. Suppression of NANOG Did Not Affect MGMT Expression

**Figure 4:**
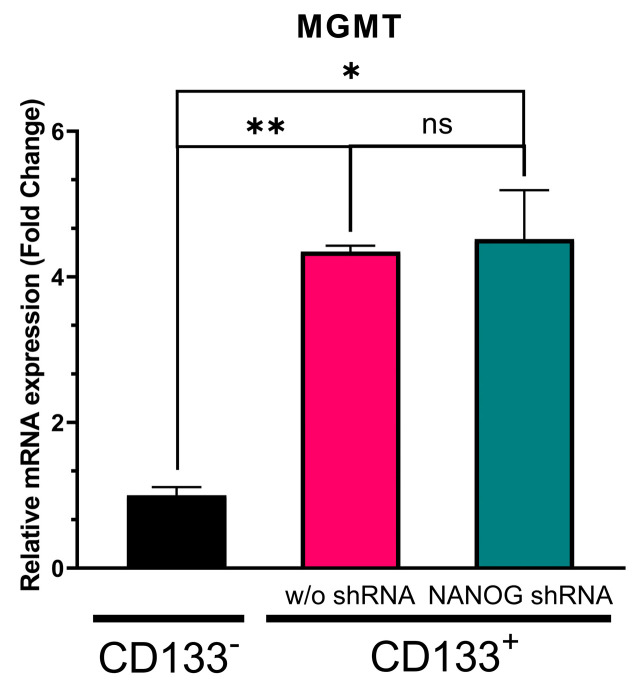
Relative MGMT transcription level in CD133^−^ GBM cells, CD133^+^ GBM cells, and CD133^+^ GBM cells treated with shRNA targeting NANOG. Relative expression was normalized using the housekeeping gene β-actin. The relative gene expression level was expressed as fold changes compared to the gene expression level in CD133^−^ cells. The statistical analysis was performed with a one-way ANOVA followed by the Holm–Sidaks multiple comparison test. * *p* < 0.05, ** *p* < 0.01.

We have previously reported that the transfer of NANOG via exosomes can potentially upregulate drug-resistance genes in GBM [26]. Therefore, we investigated the effect of NANOG suppression on a drug protection mechanism, MGMT, which is a crucial DNA repair enzyme that can reverse the effects of TMZ alkylation. Since MGMT has been shown to be elevated in CSCs [18], we expected that the suppression of NANOG expression in the CD133^+^ GBM cells would increase the efficacy of TMZ by reducing MGMT expression. We found that the MGMT transcription levels were approximately three-fold higher in the CD133^+^ GBM cells with a high NANOG expression than in the CD133^−^ GBM cells (Figure 4). However, the suppression of NANOG did not alter the MGMT expression in the CD133^+^ GBM cells. Despite the higher expressions of both NANOG and MGMT in the CD133^+^ GBM cells relative to the CD133^−^ GBM cells, this result indicates that NANOG may not directly regulate MGMT expression.

### 3.4. Effect of NANOG Suppression on PI3K/AKT Pathway

**Figure 5:**
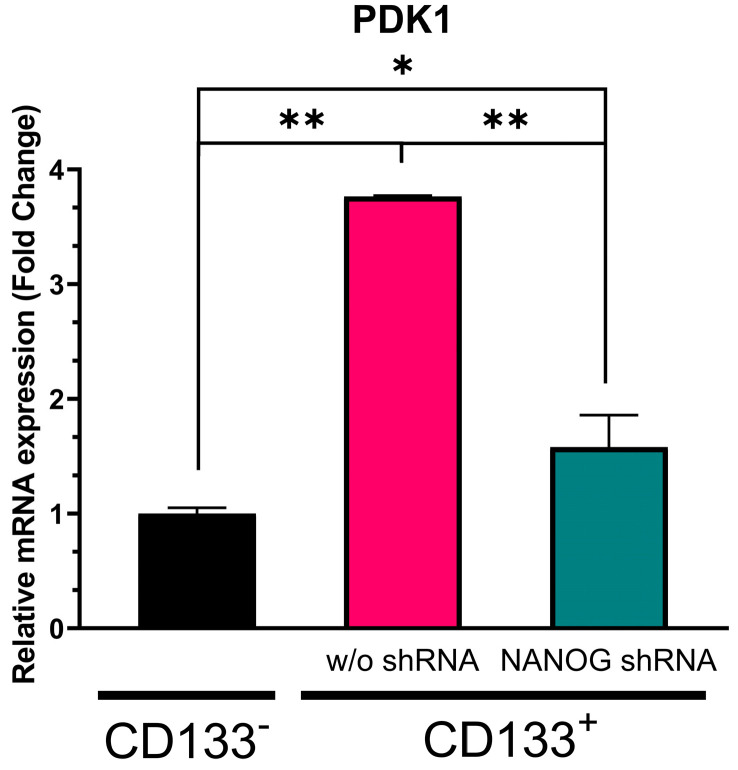
Relative mRNA expression of PDK1 in CD133^−^ GBM cells, CD133^+^ GBM cells, and CD133^+^ GBM cells treated with N-shRNA. Relative expression was normalized using a housekeeping gene, β-actin, as an internal standard. The relative gene expression level was expressed as fold changes compared to the gene expression level in CD133^−^ cells. The statistical analysis was performed with a one-way ANOVA followed by the Holm–Sidaks multiple comparison test. * *p* < 0.05, ** *p* < 0.001.

Pyruvate dehydrogenase kinase 1 (PDK1) is a crucial regulator of the PI3K/AKT pathway, which phosphorylates AKT. The promoter of PDK1 contains the nuclear factor-κB (NF-κB), and NF-κB is a binding target for NANOG [49]. As shown in Figure 5, our data demonstrated that PDK1 expression was significantly higher (more than 2.5-fold, *p* = 0.000039) in the CD133^+^ GBM cells than in the CD133^−^ GBM ones. The treatment with shRNA targeting NANOG resulted in a reduction in PDK1 levels by over 50% (*p* = 0.000082). When compared to the CD133^−^ GBM cells, the PDK1 levels were still 0.6-fold higher in the CD133^+^ GBM cells with NANOG silenced (*p* = 0.010454). NANOG expression was still significantly higher in the shRNA-treated CD133^+^ cells compared to the CD133^-^ cells, suggesting that the regulation pattern between NANOG expression and PDK1 may not be directly proportional. Although the proteomics would have to be further studied, these results may indicate a relationship between NANOG levels and PDK1 expression in GSCs, suggesting that this may act as a potential regulator of PDK1 transcriptionally.

### 3.5. Suppression of NANOG Expression Caused G0–G1 Arrest of GSCs

**Figure 6:**
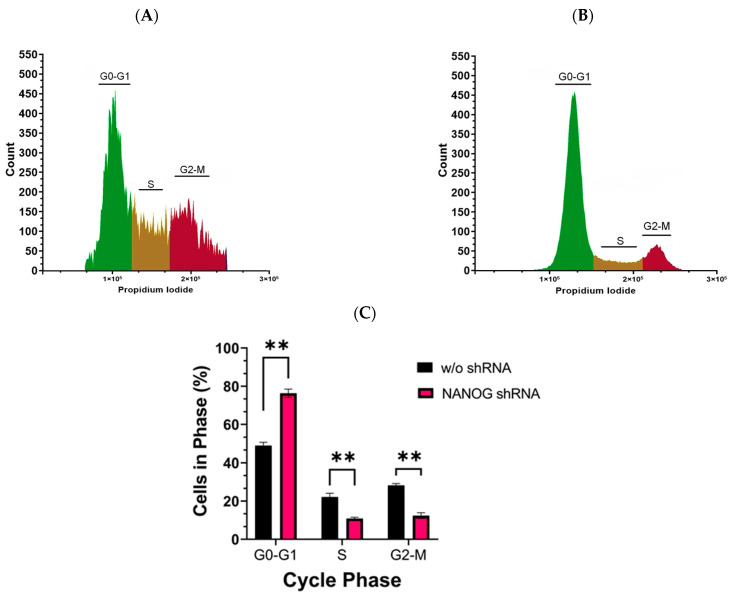
Cell cycle analysis of CD133^+^ GBM cells was performed using flow cytometry with propidium iodide staining. (**A**) represents the cell cycle analysis without shRNA targeting NANOG, while (**B**) represents the cell cycle analysis with shRNA targeting NANOG. The experiment was conducted in triplicate. The bar graph in (**C**) shows the mean and standard error (SE) of the percentage of cells in each phase of the cell cycle. Statistical analysis was performed using a multiple unpaired *t*-test, with ** indicating *p* < 0.01.

The activation of cell signaling pathways is crucial for maintaining the stem-like properties of CSCs. The PI3K/AKT pathway, particularly, plays an important role in regulating cell proliferation [35,50]. To investigate the impact of suppressing NANOG expression on the cell cycle of CD133^+^ GBM cells with reduced PDK1 levels, we performed flow cytometry analysis using propidium iodide staining. Our results revealed that the suppression of NANOG led to G0–G1 arrest in the GSCs (Figure 6). The percentage of cells in the G0–G1 phase significantly increased (76.31 ± 1.59) following the treatment with shRNA targeting NANOG, while the percentage of cells in the S (10.86 ± 1.197) and G2-M (12.36 ± 1.035) phases decreased. These findings suggest that suppressing NANOG expression inhibited the proliferation of CSCs by inducing cell cycle arrest at the G0–G1 phase.

## 4. Discussion

Glioblastoma is a highly aggressive tumor that accounts for nearly half of all CNS tumor cases. Over the past decade, the number of cases of GBM has been increasing worldwide, and this trend is expected to continue due to the growing population of individuals aged 60 and above [51]. The location of the tumor in the brain poses unique challenges for treatment compared to other types of tumors. Early diagnosis of GBM is challenging because symptoms often manifest when the disease has progressed significantly, which is similar to many other neurological disorders. Currently, there are limited diagnostic tools available to detect GBM at early stages, making early intervention difficult. By the time of diagnosis, the tumor has typically infiltrated deep into the brain, making the complete removal of the tumor tissue without damaging the surrounding healthy brain tissue nearly impossible. Additionally, any drug treatment administered must be able to cross the BBB in order to target the tumor cells within the brain effectively.

Temozolomide is the primary drug used for newly diagnosed GBM patients. Its cytotoxic effect is achieved through the methylation of the N7 position of guanine, the O3 position of adenine, and the O6 position of guanine [25]. When combined with radiation therapy, TMZ has been shown to increase patients’ lifespan [21]. However, the efficacy of the current treatments is limited, and most patients only survive for a few years even with all available treatment options explored. Patient survival rates are significantly reduced based on the level of care received [52].

The classification of GBM into proneural, neural, classical, and mesenchymal subtypes based on gene expression profiles has provided valuable insights into the molecular heterogeneity of the disease [53]. The proneural subtype is highly proliferative, while the mesenchymal subtype is slow-growing and more invasive [54]. This classification scheme has helped in understanding the diverse characteristics and behaviors of GBM tumors, and it has been associated with differences in patient prognosis and response to treatment. However, it is important to note that classifying GBM subtypes based on gene expression profiles has limitations. The classification is based on specific gene expression patterns and may not fully capture the complexity of the tumor biology or all relevant molecular alterations. Therefore, while the classification into subtypes based on gene expression profiles has provided a useful framework for understanding GBM heterogeneity, further research is needed to refine and expand our understanding of the disease. Incorporating additional molecular and genetic features, such as the expression of embryonic stem cell genes in CSCs, as well as other clinical and pathological factors, may help to improve the classification and provide a more comprehensive understanding of GBM.

Cancers that express NANOG demonstrate greater aggressiveness compared to those lacking NANOG expression [40]. NANOG-positive cells also express CD133, a well-established CSC marker, and the expression level of CD133 is reported to correlate with the pathological grade of the glioma [55,56,57]. Therefore, the population of CSCs, which is represented by NANOG expression, maybe a good way to classify GBM. Although the population of CSCs is small, they adapt to environmental changes for survival by switching states and having a high plasticity. They are the primary contributor to therapeutic resistance and could be a good target for GBM therapy.

To improve the prognosis of cancer patients, it is vital to develop therapies that can effectively eliminate CSCs, thereby preventing cancer recurrence and metastasis. As a result, there has been a growing focus on studying the biology of CSCs in order to identify effective therapeutic targets. Among the various strategies being explored, targeting CSC-specific surface markers is one approach. However, it is important to note that CSCs are known to undergo constant evolution, which can result in variations in the expression of these markers over time.

Despite efforts in a clinical trial to target EGFRvIII, which has been identified as a potential marker for CSCs in glioblastoma, no significant beneficial results were observed for the participating patients. The trial did not demonstrate the intended efficacy of the treatment in improving patient outcomes [58]. This outcome highlights the challenges and complexities involved in developing effective therapies for targeting specific molecular markers such as EGFRvIII. Another strategy was to target cell-signaling pathways that are overactive in CSCs. To inhibit notch signaling, the γ-secretase inhibitor MK-0752 has been used in several clinical trials. Although it was well tolerated, the clinical effects were minimal [59]. By inhibiting the enzymatic activity of γ-secretase, which is responsible for the cleavage of intracellular notch, researchers aimed to disrupt the signaling cascade that contributes to the maintenance and self-renewal of CSCs. The modest clinical outcomes indicate the complexities of targeting CSCs and disrupting their signaling pathways.

A promising strategy may involve targeting specific genes associated with stemness, such as NANOG and OCT4. These genes encode transcription factors that play a crucial role in maintaining the pluripotency of embryonic stem cells. Previous studies have established NANOG and OCT4 as distinguishing factors for CSCs, and the expression of these genes has been found to have detrimental effects on cancer patients [32,60,61,62]. It is well established that the promoter regions of NANOG and OCT4 contain binding sites for each other, thus facilitating their mutual expression. Additionally, these two genes regulate a significant number of overlapping genes as well as independent binding sites [63]. Recently, we found that the secretion of exosomes containing NANOG altered the transcription profile of drug-resistant genes [26]. Hence, building upon this discovery, our study aimed to specifically investigate potential targets that could be influenced by NANOG. While we did observe that the suppression of either NANOG or OCT4 heightened the sensitivity of CSCs to TMZ, our focus in the current investigation was on exploring the specific targets that could be impacted by NANOG. Considering their distinct target genes and roles in cellular processes, conducting a separate investigation into OCT4 in future studies will enable us to gain a more comprehensive understanding of its function in controlling specific pathways. By studying them independently, we can unravel their unique functions and shed light on the intricate mechanisms underlying their regulatory effects.

In lung cancer, CSCs have been shown to express high levels of OCT4, leading to increased invasiveness and resistance to chemotherapy, such as cisplatin, compared to other cancer cells [64]. Additionally, the expression of NANOGP8 has been found to promote cell migration and proliferation in gastric cancer cells [39]. A high expression of NANOG and OCT4 in renal cell carcinomas has been associated with poor patient survival, highlighting the importance of targeting these genes to improve patient outcomes [27]. It is worth noting that the expression of NANOG and OCT4 is indeed negligible in adult cells. This characteristic is advantageous when considering the potential modification of these gene expressions, as it is less likely to affect normal cells. By targeting NANOG and OCT4, we can minimize disturbances to the viability and function of normal adult cells, focusing on the therapeutic impact on cancer cells that overexpress these genes. By utilizing RNAi, we can effectively and selectively silence various genes. For instance, hyaluronan-mediated motility receptor (HMMR), an oncogene that has been found to promote stemness in GBM, when silenced with shRNA, inhibited the growth of CSCs [65]. This has also been observed with brain cytoplasmic 200 (BC200), where shRNA led to a retraction of the stemness phenotype along with the sensitization of CSCs to TMZ [66]. In our study, we selectively suppressed genes that were not expressed in normal adult cells to mitigate drug resistance in CSCs. This approach offers a safer alternative and is beneficial for the overall wellbeing of patients, as it specifically targets CSCs while potentially minimizing the impact on normal cells and reducing the side effects associated with anticancer drugs.

The findings of our current study provide compelling evidence for the potential therapeutic benefit of targeting NANOG and OCT4 in CSCs. We employed shRNA to suppress the expression of these stem cell genes, and the results were remarkable. We investigated the individual effects of silencing NANOG and OCT4 on the response of CSCs to TMZ, a commonly used chemotherapeutic agent. By using shRNA to suppress NANOG or OCT4 expression in CSCs, we observed a significant improvement in the efficacy of TMZ treatment. This suggests that the presence of NANOG and OCT4 contributes to the resistance of CSCs to TMZ, and by targeting these genes, we can sensitize CSCs to the effects of chemotherapy. The combined treatment approach resulted in a significantly greater reduction in CSC viability and an enhanced suppression of CSC-related properties compared to either treatment alone. This synergistic effect indicates that the combination of TMZ and stem cell gene silencing acts through complementary mechanisms, leading to a more profound and comprehensive targeting of CSCs.

Our recent discoveries have revealed the presence of NANOGP8 in the exosomes released by cancer cells [34]. These exosomes have been found to impact the transcriptional profile of several drug-resistant genes in GBM [26]. Notably, one of the genes that displayed an altered transcriptional activity was MGMT. Consequently, our study aimed to investigate how NANOG influences drug-resistant genes in GSCs. MGMT plays a crucial role in the neutralization of TMZ by removing alkyl groups from guanine residues. Consistent with prior research, our findings corroborated that MGMT gene expression is significantly higher in GBM CSCs compared to non-CSC GBM cells (CD133^−^). However, intriguingly, when we suppressed NANOG expression, we did not observe any noticeable changes in MGMT expression levels. This suggests that NANOG may regulate drug resistance to TMZ treatment in GSCs through mechanisms other than the direct upregulation of MGMT.

The PI3K/AKT pathway significantly regulates the cell cycle by promoting cell proliferation and inhibiting apoptosis. The hyperactivation of the pathway has been shown to create drug resistance in cancer [50], which is particularly prevalent in GBM, and therapeutic strategies targeting this pathway have been explored [67,68].

Our current study demonstrated that when NANOG expression was suppressed in CSCs, there was a notable decrease in PDK1 gene expression. Silencing PDK1 mRNA expression reduces the activity of the PI3K/AKT pathway, and elevated PDK1 mRNA expression drives the PI3K/AKT pathway [69,70]. These observations underscore the critical role of PDK1 as a regulator of the PI3K/AKT pathway, specifically in modulating the phosphorylation of AKT. The observed reduction in PDK1 expression levels upon suppressing NANOG suggests that NANOG may play a role in modulating AKT phosphorylation and, consequently, regulating the PI3K/AKT pathway. Our study specifically examined the relationship between NANOG and PDK1, shedding light on their interaction within the pathway. By influencing PDK1 expression, NANOG has the potential to exert control over AKT phosphorylation and activation, thereby impacting downstream signaling events in the pathway.

However, further research is required to elucidate the precise mechanisms through which NANOG regulates PDK1 and understand its downstream effects on the PI3K/AKT pathway. To gain a more comprehensive understanding of the PI3K/AKT pathway and its modulation by NANOG, it is crucial to investigate the involvement of other factors, both upstream and downstream, that interact with PDK1. This broader assessment would provide a more complete picture of the molecular interactions within the pathway and the potential influence of NANOG on these interactions. Continued research in this area will contribute to a deeper understanding of the molecular mechanisms underlying the role of NANOG and its associated signaling pathways in CSCs, ultimately guiding the development of novel therapeutic approaches.

Upon suppressing NANOG expression, we observed a G0/G1 cell cycle arrest in CSCs, indicating that NANOG plays a crucial role in regulating cell proliferation. It is well known that AKT activation facilitates cell cycle progression, particularly by promoting the transition from the G1 phase to the S phase. Conversely, inhibiting AKT activity can induce G0/G1 cell cycle arrest, thus preventing cells from entering the S phase and undergoing DNA replication [71]. Additionally, inhibiting the PI3K/AKT pathway can lead to the upregulation of cyclin-dependent kinase inhibitors, such as p21 and p27, which are known to induce G1 cell cycle arrest [72]. These observations emphasize the significant role of the PI3K/AKT pathway in governing the cell cycle, particularly the transition from the G0/G1 phase.

Understanding the intricate interplay between NANOG, the PI3K/AKT pathway, and cell cycle regulation provides valuable insights into the mechanisms underlying cell proliferation in CSCs. Although further investigation on other signaling pathways to increase drug resistance in CSCs may be needed, these insights hold promise for deepening our understanding of drug resistance mechanisms and identifying potential therapeutic targets to eliminate CSC, which can lead to the cure of GBM.

## 5. Conclusions

Extensive research on CSCs has been conducted in the field of cancer research, as they share many characteristics with normal stem cells, although their origin is unclear. To achieve a successful treatment for cancers such as GBM, it is essential to develop therapies that can effectively target and eliminate CSCs. Embryonic stem cell genes play a pivotal role in regulating the stem-like properties of CSCs and contribute significantly to therapeutic resistance.

Our study demonstrated that suppressing the expression of stem cell genes NANOG and OCT4 can enhance the effectiveness of TMZ treatment in CSCs derived from GBM. Our findings suggest that this improvement may be attributed to the downregulation of the PDK1 gene by NANOG suppression, resulting in reduced AKT phosphorylation within the PI3K/AKT pathway. Consequently, this may lead to G0/G1 cell cycle arrest and increased susceptibility to chemotherapy. These results provide a foundation for considering NANOG and OCT4 as viable gene targets for GBM treatment. Notably, these factors are not expressed in adult cells, allowing for the selective targeting of CSCs without side effects. The use of shRNA to suppress these embryonic stem cell genes combined with existing treatment strategies holds great potential for clinical applications to improve patient outcomes significantly.

Further investigations using multiple primary cell samples are necessary to unravel the underlying molecular mechanisms in detail and translate these research findings into practical clinical applications for the benefit of cancer patients. Continued research in this area will contribute to advancing personalized medicine and improving treatment outcomes for individuals affected by GBM and other cancers.

## Figures and Tables

**Table 1:** A list of qPCR primers.

Primer	Sequence
NANOG-F	5′-GTCTTCTGCTGAGATGCCTCACA-3′
NANOG-R	5′-TCTGCTGGAGGCTGAGGTAT-3′
OCT4-F	5′-GGAAGGTATTCAGCCAAACGACCA-3′
OCT4-R	5′-CTCACTCGGTTCTCGATACTGGTT-3′
MGMT-F	5′-TTCACCATCCCGTTTTCCAG-3′
MGMT-R	5′-ATTGCCTCTCATTGCTCCTC-3′
PDK1-F	5′-TCGTCCTCCTCCTCACACTCCCT-3′
PDK1-R	5′-GCCTGCTTCTCCAACAACAACCTCTT-3′
B-Actin-F	5′-AGAGCTACGAGCTGCCTGAC-3′
B-Actin-R	5′-AGCACTGTGTTGGCGTACAG-3′

**Table 2:** CDI values for TMZ and shRNA treatment on CD133^+^ GBM.

Combination of TMZ and shRNA	CDI
TMZ 10 µM + shRNA NANOG	0.25
TMZ 100 µM + shRNA NANOG	0.26
TMZ 1000 µM + shRNA NANOG	0.28
TMZ 10 µM + shRNA OCT4	0.2
TMZ 100 µM + shRNA OCT4	0.18
TMZ 1000 µM + shRNA OCT4	0.21

## Data Availability

The data presented in this study are available in the article. The original raw data are available on request from the corresponding author.
